# Leptomeningeal metastasis from colon cancer: a case report and review of literature

**DOI:** 10.3389/fonc.2026.1935585

**Published:** 2026-09-03

**Authors:** Chao Wang, Daixi Wu, Aolin Wang, Xiaojia Zhang, Jianghui Duan, Mingrui Dong, Mingxiao Li, Zhirong Qi, Huijuan Cui

**Affiliations:** 1 Department of Integrative Oncology, China-Japan Friendship Hospital, Beijing, China; 2 Clinical Medical College of Traditional Chinese Medicine, Capital Medical University, Beijing, China; 3 China-Japan Friendship Clinical Medical College, Beijing University of Chinese Medicine, Beijing, China; 4 Department of Diagnostic Radiology, China-Japan Friendship Hospital, Beijing, China; 5 Department of Neurology, China-Japan Friendship Hospital, Beijing, China; 6 Department of Neurosurgery, China-Japan Friendship Hospital, Beijing, China

**Keywords:** case report, colon cancer, leptomeningeal metastasis, meningeal carcinomatosis, prognosis, signet-ring cell carcinoma

## Abstract

**Background:**

Leptomeningeal metastasis from colorectal cancer (LM-CRC) is a rare condition characterized by poor outcomes and no established standard of care.

**Case description:**

We report a 60-year-old female patient who presented with constipation and abdominal pain. Eight months prior, she had been diagnosed with poorly differentiated adenocarcinoma of the sigmoid colon with focal signet-ring cell features, proficient mismatch repair, and a KRAS mutation. She had received treatment with FOLFOX/FOLFIRI ± bevacizumab. Five days before admission, she developed headache and vomiting. Contrast-enhanced brain MRI suggested leptomeningeal metastasis, which was confirmed by lumbar puncture cytology. Owing to progressive symptom deterioration, the patient did not receive any antitumor therapy timely and died two weeks after symptom onset. In this review, we summarize 29 cases of LM-CRC, aiming to characterize the pathology, symptoms, diagnosis, and survival data of LM-CRC in order to enhance clinicians’ awareness of this rare disease.

**Conclusion:**

The incidence of LM-CRC is low and the prognosis is extremely poor; active anti-tumor therapy may prolong patient survival.

## Introduction

Leptomeningeal metastases (LM) are a rare and advanced complication, most often occurring in patients with breast cancer, non-small cell lung cancer, melanoma, and hematologic malignancies (1). They affect approximately 5–10% of patients with solid tumors and 5–15% of those with hematologic cancers (2, 3). However, the case series from MD Anderson and Mayo Clinic demonstrated that LM from gastrointestinal malignancies are extremely uncommon, with an incidence of 0.019%–0.058% (3, 4). This study describes the case of an elderly woman with poorly differentiated sigmoid adenocarcinoma, including focal signet-ring cell features, who developed LM. Despite her primary tumor being stable, she experienced rapid clinical decline and death.

We conducted a comprehensive PubMed search using a combination of MeSH terms and free-text keywords: ((((“Meningeal Carcinomatosis”[Mesh]) OR (meningeal metastasis)) OR (leptomeningeal carcinomatosis)) OR (Neoplastic Meningitis)) AND (((“Colorectal Neoplasms”[Mesh]) OR (colon cancer)) OR (rectal cancer)). The search period was from database inception to April 16, 2026. We identified 84 studies. After screening titles and abstracts, relevant studies on CRC and LM were selected for full-text review, and the reference lists of key articles were also examined. We obtained a total of 28 case reports and, based on the collected evidence, provided an up-to-date overview of the mechanisms, clinical manifestations, diagnosis, treatment, and prognosis of LM-CRC (**Table 1**).

**Table 1:** Summary of case reports of LM-CRC.

Patient	Pathologic type	Publication year	Diagnostic method	CSF analysis	Neurological symptoms/signs	Treatment for LM	Time to death from symptom onset wk
1 current case	Adenocarcinoma with signet-ring cell differentiation	—	CSF	tumor cells	headache, vomiting, seizures	——	2
2 (5)	Adenocarcinoma with signet-ring cell differentiation	2021	CSF	tumor cells	headache, dizziness	——	10
3 (6)	Neuroendocrine carcinoma	2019	CSF	tumor cells	facial palsy	corticosteroids	1
4 (7)	Neuroendocrine carcinoma	2018	CT	NA	seizures	CyberKnife radiosurgery	10
5 (8)	Signet-ring cell carcinoma	2017	MRI, CSF	tumor cells	headache, vomiting, diplopia	chemotherapy	——
6 (9)	Adenocarcinoma	2015	CSF	tumor cells	headache	ventriculoperitoneal shunt	4
7 (10)	Adenocarcinoma with signet-ring cell differentiation	2013	MRI, CSF	tumor cells	headache	radiotherapy	6
8 (11)	Adenocarcinoma	2012	MRI, CSF	tumor cells	decreased hearing, dizziness, facial palsy, gait disturbance, dysarthria, dysphagia	——	6
9 (12)	Adenocarcinoma	2011	MRI, CSF	Elevated CEA and protein	ataxia, dysarthria	——	2
10 (13)	Unspecified	2009	CT	NA	dizziness, gait disturbance	——	4
11 (14)	Adenocarcinoma with signet-ring cell differentiation	2006	MRI, CSF, Autopsy	tumor cells	dyspnea, confusion	——	2
12 (15)	Unspecified	2024	MRI, CSF	tumor cells	headache, nausea or vomiting, dizziness, confusion	——	——
13 (16)	Signet-ring cell carcinoma	2022	CSF	Elevated protein	facial palsy, limb weakness, dysarthria	radiotherapy and chemotherapy	——
14 (17)	Adenocarcinoma	2024	MRI, CSF	tumor cells, Elevated CEA	lethargy, limb weakness	corticosteroids	2
15 (18)	Adenocarcinoma with signet-ring cell differentiation	2022	MRI, Autopsy	Elevated CEA	headache, nausea, dizziness, dysarthria, neck pain	——	1
16 (19)	Unspecified	2020	CSF	tumor cells, ElevatedCA 199	headache, vomiting	chemotherapy	6
17 (20)	Adenocarcinoma	2020	MRI, Surgical pathology	NA	limb paresthesia, back pain	operation	21
18 (21)	Signet-ring cell carcinoma	2018	MRI	NA	diplopia	——	3
19 (22)	Unspecified	2017	CSF	tumor cells	NA	chemotherapy	——
20 (23)	Signet-ring cell carcinoma	2015	MRI, CSF	tumor cells	decreased hearing, ataxia	radiotherapy	6
21 (24)	Adenocarcinoma	2015	MRI	NA	neck and limb pain	operation	——
22 (25)	Unspecified	2010	CSF	tumor cells, Elevated CEA	decreased hearing and vision	radiotherapy	——
23 (26)	Signet-ring cell carcinoma	2010	CSF	tumor cells	headache, decreased hearing and vision	radiotherapy	8
24 (27)	Adenocarcinoma	2006	CSF	tumor cells	decreased hearing, facial palsy	——	16
25 (28)	Adenocarcinoma	1995	MRI, CSF	tumor cells, Elevated CEA	headache, nausea or vomiting	radiotherapy and chemotherapy	2
26 (29)	Signet-ring cell carcinoma	1994	Autopsy	NA	headache, nausea, decreased vision	——	3
27 (30)	Adenocarcinoma	1979	CSF	tumor cells	headache	radiotherapy and chemotherapy	12
28 (31)	Signet-ring cell carcinoma	2017	MRI, CSF	tumor cells	decreased vision, confusion	radiotherapy	——
29 (32)	Adenocarcinoma	2001	MRI, CSF	tumor cells	Nausea and headache	——	4

## Case presentation

**Figure 1:**
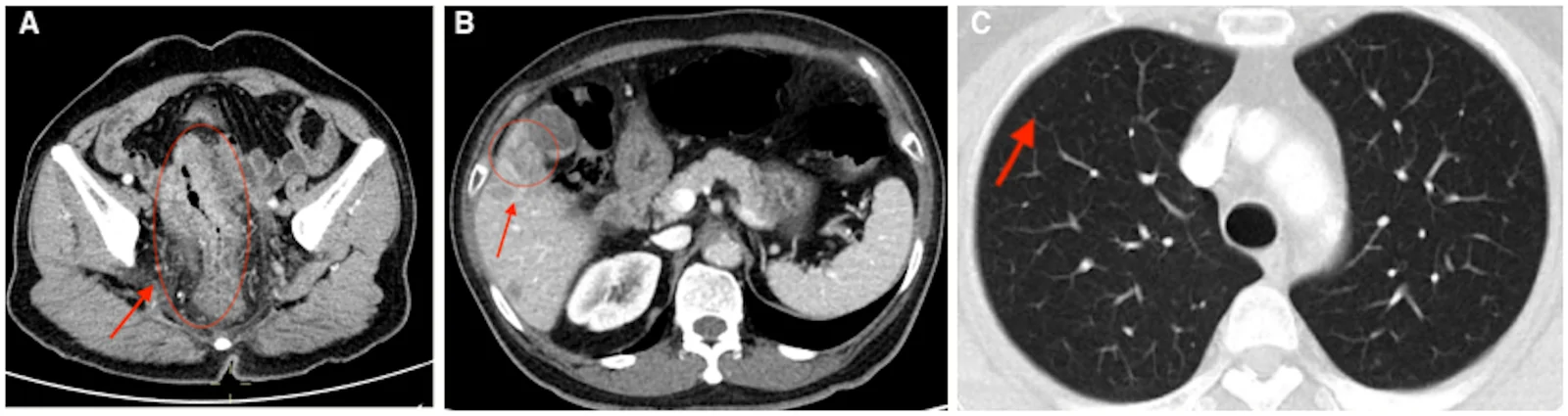
Imaging at initial diagnosis (July 2025). **(A)** Sigmoid colon (oblique axial view); **(B)** Liver metastasis; **(C)** Small pulmonary nodule.

**Figure 2:**
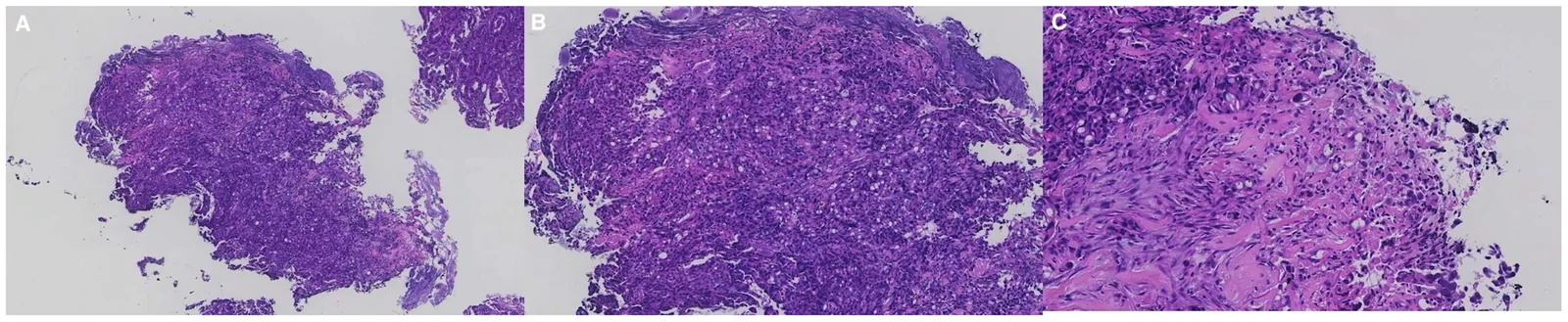
Pathology at initial diagnosis (July 2025). **(A)** Poorly differentiated adenocarcinoma (HE stain, 100×); **(B)** Poorly differentiated adenocarcinoma (HE stain, 200×); **(C)** Signet-ring cell carcinoma (HE stain, 400×).

A 60-year-old female patient was admitted to our hospital because of intermittent abdominal pain, a diagnosis of colorectal cancer for 8 months, and headache with vomiting for 5 days. Eight months prior, the patient experienced abdominal pain. Colonoscopic pathology revealed poorly differentiated adenocarcinoma of the sigmoid colon with focal signet-ring cell features. Contrast-enhanced CT revealed an ill-defined tumor with surrounding infiltration, multiple enlarged peritumoral and peritoneal lymph nodes, as well as multiple liver metastases and several tiny pulmonary nodules that could not be definitively excluded as metastases (**Figures 1**, **2**). These findings were consistent with clinical stage cT4aN2aM1b (stage 4). Mismatch repair proteins (MLH1, MSH2, MSH6, PMS2) were preserved. Molecular analysis identified a KRAS exon 2 mutation (G12S or G12D). The liver and lung metastases increased in size and number, with an evaluation of progressive disease (PD) after 6 cycles of FOLFOX ± bevacizumab. Subsequently she received 7 cycles of FOLFIRI + bevacizumab. Five days ago, the patient developed headache and vomited > 10 times. Therefore, the patient came to our unit for medical treatment. The patient has a 40-year history of chronic eczema and a 10-year history of diabetes mellitus, and denies any history of chronic conditions such as hypertension or cardiovascular disease.

Nuchal rigidity was present. Upper limb muscle strength was grossly intact. Lower limb muscle strength was grade 4. Tendon reflexes were present. No pathological reflexes were elicited. No obvious abnormalities of the cranial nerves were observed.

Complete blood count, liver and kidney function tests, coagulation profiles, and tumor markers showed no significant abnormalities. Routine cerebrospinal fluid (CSF) examination revealed no abnormalities. CSF biochemistry showed: glucose 0.1 mmol/L (reference range: 2.24–4.2), lactate dehydrogenase 389 U/L (100–250), microalbumin 960.2 mg/L (140–230), chloride 115.6 mmol/L (120–132), total protein 1.057 g/L (0.05–0.45), and adenosine deaminase <4 U/L (4–24). India ink staining, bacterial smear, acid-fast bacillus smear, and next-generation sequencing of the CSF were all negative. Autoimmune encephalitis antibody panel and paraneoplastic antibody panel were also negative. A lumbar puncture detected malignant cells.

**Figure 3:**
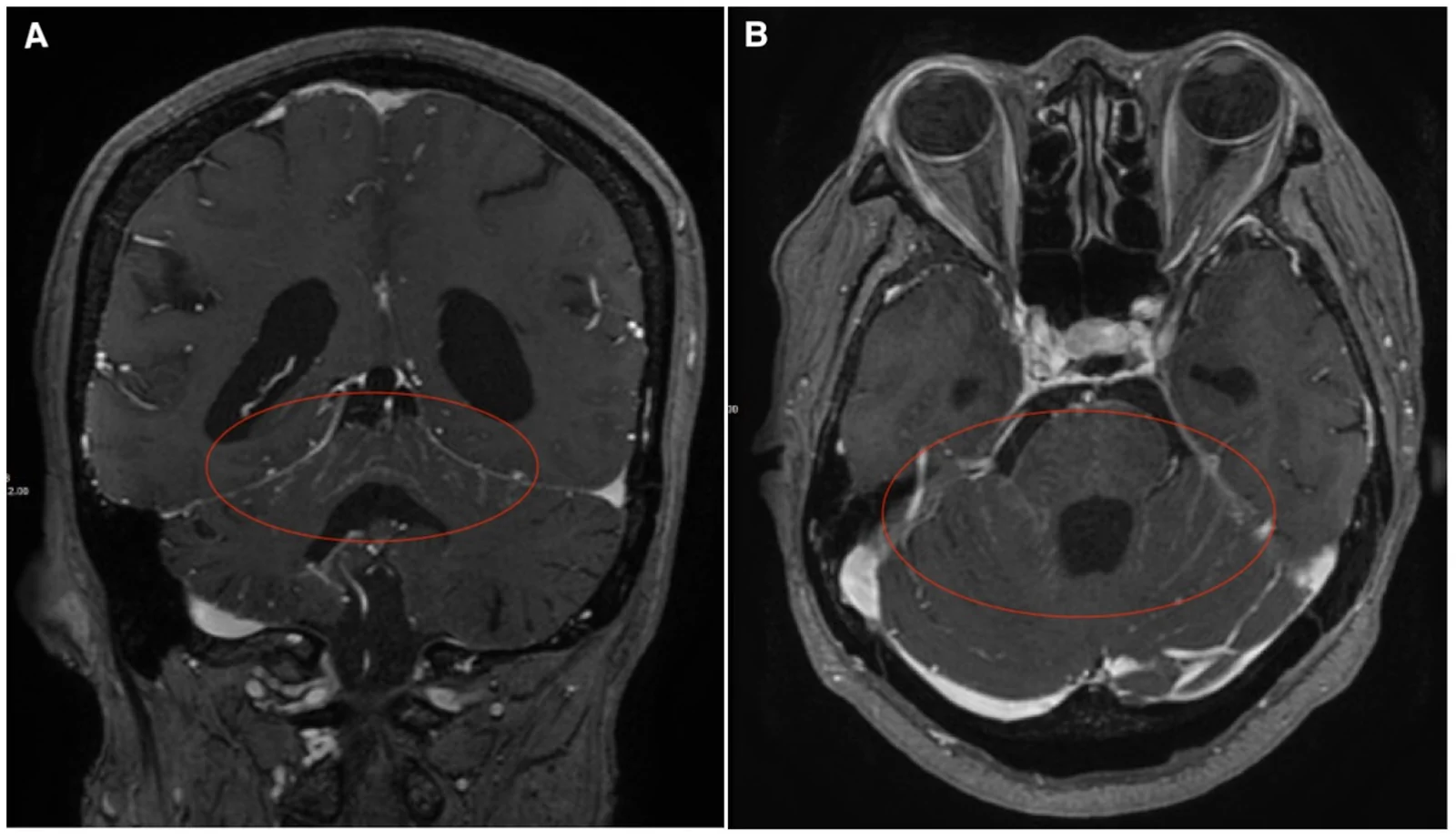
Leptomeningeal metastasis images (March 2026). **(A)** Linear thickening of the cerebellar leptomeninges (coronal view); **(B)** Axial view.

As shown in **Figure 3**, contrast-enhanced brain MRI demonstrated linear thickening of the leptomeninges over the bilateral cerebellum, without clear evidence of parenchymal metastases.

**Figure 4:**
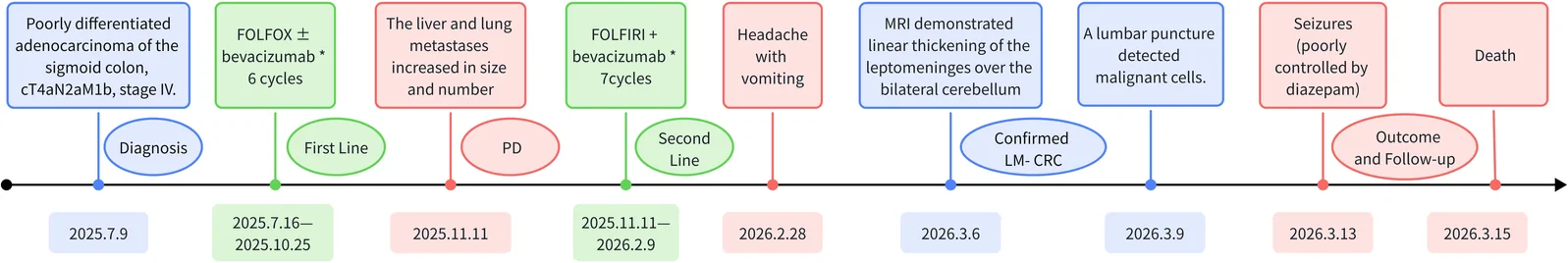
A timeline with relevant data from the episode of case.

The final diagnosis was LM from sigmoid colon adenocarcinoma. The patient was scheduled to undergo brain radiotherapy at another hospital. However, on the second day after transfer to the other hospital, the patient developed seizures (poorly controlled by diazepam) and died on the fourth day without timely treatment. The time from the onset of neurological symptoms to death was 2 weeks. Overall survival (OS) from the diagnosis of colorectal cancer was 8 months, and survival from the diagnosis of LM by brain MRI was 8 days. A timeline of the case is shown in **Figure 4**.

## Discussion

### Mechanisms

Cancer cells may gain entry to the leptomeningeal space through a number of routes, among which hematogenous spread, perineural infiltration, and indirect secondary metastasis are the major pathways (33). (1) Arterial dissemination: Tumor cells may enter the choroid plexus stroma (the specific mechanism remains unclear), inciting a local inflammatory response to disrupt the endothelial barrier and loosen the tight junctions of choroid plexus epithelial cells, thereby invading the CSF. (2) Perineural invasion: Cancer cells can migrate along the perineural spaces of cranial nerves, spinal nerves, and peripheral nerves to access the leptomeninges. However, this route may be most clinically relevant only when the distance that cancer cells must travel to reach the central nervous system is relatively short, such as in head and neck tumors (along cranial nerves) and spinal tumors (along spinal nerve roots). (3) Indirect secondary metastasis: Cancer cells may also invade the leptomeninges secondarily from other metastatic sites. The bone marrow and brain parenchyma represent the two major metastatic sites that enable access to the leptomeninges. Bridging vessels connect the calvarial bone marrow (located in the skull) and vertebral bone marrow (located in the spine) with the subarachnoid space. Leukemic cells express high levels of integrin α6, mediating interactions with laminin expressed on endothelial cells of these bridging vessels, thereby promoting tumor cell migration (34). Solid tumors that frequently spread to the bone marrow may also express integrin α6 or other immune-like phenotypes, enabling colonization of the CSF via vertebral and calvarial bone marrow.

### Symptoms

The clinical presentation of LM varies with the location and extent of involvement (35). Cranial nerve dysfunction is common, often involving nerves II, III, IV, V, VI, VII, and VIII, and can manifest as diplopia, facial numbness or pain, facial weakness, hearing loss, and visual impairment (36). Involvement of the spinal cord or nerve roots typically causes back or neck pain, limb paresthesias, and lower motor neuron weakness. When the cerebral hemispheres or cerebellum are involved, patients may develop confusion, cognitive decline, or ataxia. Furthermore, impaired CSF circulation may lead to increased intracranial pressure, manifesting as headache, nausea, and vomiting.

In this study, we summarized the symptoms from 29 cases of LM-CRC. Typical manifestations included headache (44.8%, 13/29), nausea or vomiting (27.6%, 8/29), diplopia or decreased vision (24.1%, 7/29), and dizziness (17.2%, 5/29). Other reported symptoms included facial palsy, deafness, seizures, neck and back pain, paresthesia of the limbs, confusion, and so on.

### Diagnosis

Contrast-enhanced magnetic resonance imaging (MRI) is the most sensitive imaging modality for LM, with substantially higher sensitivity and specificity than computed tomography (37). Typical findings include leptomeningeal enhancement and small subarachnoid nodules (38). Despite advances in imaging, CSF cytology remains the diagnostic gold standard. Diagnosis was confirmed in 25 cases by CSF cytology or autopsy; 14 of these also showed corresponding imaging abnormalities. The remaining 4 cases were diagnosed solely based on characteristic imaging findings.

However, false-negative results are common, with up to 46% of cases testing negative on the first lumbar puncture (39). Factors contributing to false negatives include low tumor burden, inadequate CSF volume (less than 5–10 mL), and delays in sample processing leading to cellular degradation (40). To reduce false-negative results, a structured approach has been proposed (41): obtaining an adequate CSF volume (at least 10.5 mL), ensuring prompt laboratory processing, collecting CSF near known or suspected sites of disease, and repeating lumbar puncture if initial cytology is negative. The sensitivity of a single lumbar puncture is approximately 70%, increasing to 80–85% with a second procedure; further sampling generally provides limited additional benefit.

CSF tumor markers may provide additional diagnostic support. As the blood–brain barrier restricts the passage of large molecules, markers such as carcinoembryonic antigen (CEA) are normally present at very low levels in CSF. An elevated CSF CEA (>1 ng/mL) in the setting of a relatively low serum CEA (<100 ng/mL) strongly suggests LM (42). When CSF CEA exceeds serum levels, this indicates local production within the central nervous system and can support the diagnosis even if cytology is negative.

Emerging liquid biopsy techniques show promise for improving diagnostic accuracy in LM (43, 44). For instance, rare cell capture technology for detecting CSF circulating tumor cells (CTCs) has demonstrated high sensitivity and specificity, even at a low threshold of 1 CTC/mL (41). Beyond detection, CSF CTC counts can indicate early disease activity, sometimes increasing months before MRI changes or positive cytology (45, 46). Epigenetic markers are also under investigation. SHP1P2, an epithelial-specific methylation marker (previously studied in non-small cell lung cancer), has shown utility in distinguishing epithelial-derived malignancies with LM from benign meningeal conditions or malignancies without LM (47). Its reported high sensitivity and specificity suggest it could be a valuable diagnostic adjunct for LM.

### Treatment

Given the extremely poor prognosis of LM, treatment is primarily palliative. While multimodal approaches offer the best outcomes, their benefits remain limited. Treatment approaches among the 29 patients varied: 15 patients did not receive antitumor therapy (12 received no treatment, 2 were treated with corticosteroids for symptomatic relief, and 1 underwent ventriculoperitoneal shunting for decompression). Five patients received radiotherapy alone, three received chemotherapy alone, three received combined radiotherapy and chemotherapy, and three underwent surgical intervention. Currently, no standard treatment exists specifically for LM-CRC; most strategies adapt therapies used for brain metastases or LM in other malignancies, such as intrathecal chemotherapy and radiotherapy (48).

Intrathecal therapy: Intrathecal (IT) administration is most effective in patients with unobstructed CSF flow and thin, linear meningeal involvement. Its limited penetration into bulky disease reduces efficacy in nodular lesions thicker than 2 mm. Common agents include methotrexate, cytarabine, and thiotepa (1), which show comparable efficacy in LM from solid tumors, with no clear evidence favoring one (49–51). Several alternative agents have also been investigated. In a phase II study of 27 LM patients, intrathecal etoposide showed modest activity; 26% achieved CSF malignant cell clearance and stabilization or improvement of neurological symptoms (52). Toxicities primarily included transient chemical arachnoiditis and chronic fatigue. The same group also evaluated intrathecal alpha-interferon (α-IFN), which showed higher antitumor activity, with 45% achieving similar clinical and cytologic responses (53). However, this benefit came with substantial toxicity, particularly chronic fatigue syndrome, significantly affected quality of life. Another study of 23 LM patients assessed intrathecal 5-fluoro-2’-deoxyuridine (FdUrd); all patients reported relief from headache and neck pain, and 70% achieved a complete or PR without significant neurotoxicity (54).

Radiotherapy: Radiotherapy remains an important component of LM management. Photon involved-field radiotherapy (IFRT) is typically used for bulky disease or when CSF flow is obstructed (55). Given the diffuse nature of LM, IFRT is primarily palliative, commonly delivered as 20 Gy in 5 fractions or 30 Gy in 10 fractions, with limited overall effectiveness (56). Craniospinal irradiation (CSI) has the potential for broader disease control but is rarely used due to substantial systemic toxicity and the risk of leukoencephalopathy (55). Proton beam CSI offers a more targeted approach, delivering radiation with greater precision and reduced exposure to surrounding tissues, which may improve safety while maintaining therapeutic benefit (57, 58). A phase II study in patients with LM secondary to non–small cell lung and breast cancers demonstrated that proton CSI significantly improved central nervous system progression-free survival and OS compared with IFRT, without elevating the incidence of severe treatment-related adverse events (59).

### Prognosis

#### Active antitumor therapy and prognosis

Survival from symptom onset to death was reported or estimable for 22 cases among 29 cases, while 7 cases lacked this information. Therefore, 22 evaluable cases were included in the survival analysis and divided into two subgroups based on whether the patients received active antitumor therapy. The median OS for the non-active antitumor therapy group was 3 weeks (95% CI: 1.19–4.82), compared to 6 weeks (95% CI: 1.84–10.16) for the active antitumor therapy. This difference reached statistical significance (log-rank P = 0.042). Notably, the subgroup analysis based on treatment status is subject to severe selection bias. We must acknowledge that the conclusions are limited and should be interpreted only as exploratory in nature. While active antitumor treatment may offer a modest survival benefit, management decisions for LM-CRC should not be solely based on survival duration. Given the severe symptom burden and limited life expectancy, treatment must prioritize symptom control, focusing on pain relief and preservation of neurological function. When disease-directed therapy no longer provides meaningful benefit, early integration of palliative care becomes essential to maintain patient comfort and dignity.

#### Pathological type and prognosis

It is noteworthy that among the pathological types of LM-CRC, signet-ring cell carcinoma accounted for a high proportion. In 29 case reports, there were 6 cases (20.7%) of signet-ring cell carcinoma and 6 cases (20.7%) of adenocarcinoma with signet-ring cell differentiation, which was far higher than the proportions in CRC (1.0% and 2–5%, respectively) (60). This phenomenon was also observed by MD Anderson Cancer Center (3). This suggested that signet-ring cell differentiation might be a key driver of LM. Signet ring cell carcinoma is a subtype associated with aggressive behavior and poor outcomes. The lack of normal cell–cell adhesion in these tumor cells facilitates diffuse infiltration, vascular invasion, lymph node involvement, and widespread metastasis (61). Consequently, signet ring cell carcinoma is associated with higher rates of recurrence, peritoneal dissemination, and lower rates of curative resection compared with conventional colorectal adenocarcinoma (62). 22 evaluable cases were grouped by histology into three categories: tumors with a signet ring cell component (n=9); adenocarcinomas without a signet ring cell component(n=9); other types (n=4; 2 with unspecified pathology and 2 neuroendocrine carcinomas). The result showed the median OS was 4 weeks (95% CI: 1.08–6.92) for adenocarcinoma, 3 weeks (95% CI: 1.54–4.46) for the signet ring cell component, and 4 weeks (95% CI: 0–8.90) for other diagnoses. No statistically significant differences were observed among these groups (P = 0.397). Despite this apparent predisposition, our analysis found no statistically significant difference in survival between patients with conventional adenocarcinoma and those with signet ring cell carcinoma. This might be attributed to the limited sample size or could indicate that, once LM develops, the disease follows a uniformly aggressive course irrespective of the histologic subtype.

#### Compared with solid tumors

For patients with LM from solid tumors, median survival is approximately 4–6 weeks without treatment and may extend to 3–6 months with active therapy. Outcomes for LM-CRC appear even poorer. A study at the MD Anderson Cancer Center reported a median survival of 7 weeks with treatment and only 1 week without. In our cohort, median survival was 6 weeks for patients receiving active antitumor therapy and 3 weeks for those who did not, both notably shorter than the typical survival observed in LM from solid tumors overall. One factor that may contribute to this unfavorable prognosis is that effective systemic therapies capable of penetrating the blood–brain barrier are lacking in CRC. In contrast, targeted agents such as osimertinib and lorlatinib have improved outcomes in non–small cell lung cancer with specific genetic alterations, and combinations like neratinib or lapatinib plus capecitabine have demonstrated meaningful intracranial activity in HER2-positive breast cancer. CRC lacks comparable treatment options because commonly used targeted therapies, such as cetuximab and bevacizumab, have limited penetration into the central nervous system.

### Limitation

As a case report, the level of evidence of this study is inherently low. Although we employed a comprehensive search strategy combining MeSH terms and free-text keywords, some case reports published in non-English journals or not indexed in PubMed may have been missed, and studies whose full text was not accessible via PubMed were also not included. Among the 29 case reports, several had incomplete information on pathological type, survival time, or other variables, which limited deeper statistical analyses. Under these circumstances, we must acknowledge that the survival analysis is fragile and this part is exploratory and hypothesis-generating only. Some subgroups may have been underpowered to detect true differences due to the small sample size. Despite these limitations, given the extreme rarity of LM-CRC, this study still provides the most up-to-date and comprehensive overview of the literature to date.

## Conclusion

This study presented a rare case of a patient with colon adenocarcinoma featuring signet ring cell differentiation. The patient rapidly developed headache and vomiting, leading to an LM diagnosis, and she succumbed two weeks after symptom onset. Our review of 29 LM-CRC cases revealed a markedly higher proportion of signet ring cell differentiation compared to the general CRC population. This suggests that this histological subtype may be associated with an increased risk of LM and needs to be closely monitored in clinical practice. While active treatment may modestly extend median survival from approximately 3 to 6 weeks, management decisions must be guided by patient performance status and treatment tolerance. Consequently, palliative and supportive care remains an appropriate and crucial approach in many cases, focusing on comfort and quality of life.

## Data Availability

The original contributions presented in the study are included in the article/supplementary material. Further inquiries can be directed to the corresponding authors.
